# Two decades of targeted pulmonary artery banding in pediatric dilated cardiomyopathy

**DOI:** 10.1016/j.xjon.2026.102088

**Published:** 2026-08-28

**Authors:** Thushiha Logeswaran, Blanka Steinbrenner, Dietmar Schranz, Sabine Recla, Karin Klingel, Matthias Müller, Kerstin Gummel, Lucie Rüblinger, Bettina Reich, Martin Farr, Christian Jux, Hakan Akintürk

**Affiliations:** a Department of Pediatric Cardiology, Intensive Care Medicine and Congenital Heart Disease, Pediatric Heart Center, Justus-Liebig-University, Giessen, Germany; b Department of Pediatric Cardiology, Goethe University, University Hospital Frankfurt, Frankfurt, Germany; c Cardiopathology, Institute for Pathology and Neuropathology, University Hospital Tübingen, Tübingen, Germany; d Department of Pediatric Cardiac Anesthesiology, Pediatric Heart Center, Justus-Liebig-University, Giessen, Germany; e Department of Pediatric Cardiology, German Heart Center Munich, Technical University of Munich, Munich, Germany; f Department of General and Interventional Cardiology/Angiology, Ruhr University Bochum, Heart- and Diabetescenter NRW, Bad Oeynhausen, Germany; g Department of Pediatric Cardiac Surgery and Congenital Heart Disease, Pediatric Heart Center, Justus-Liebig-University, Giessen, Germany

**Keywords:** dilated cardiomyopathy, pulmonary artery banding, restrictive atrial septal defect, myocardial recovery, mechanical circulatory support, heart transplantation

## Abstract

**Objectives:**

Dilated cardiomyopathy (DCM) with severe left ventricular (LV) dysfunction in infants and young children is a life-threatening condition and carries a high risk of mechanical circulatory support and heart transplantation (HTx). Pulmonary artery banding (PAB) has been proposed as a recovery strategy, but data on medium- and long-term outcomes remain limited.

**Methods:**

This retrospective, single-center observational study included infants and young children who underwent surgical PAB between 2006 and 2024. Inclusion criteria were ROSS functional class (FC) III/IV, spherical left ventricular-dilated cardiomyopathy (LV-DCM) with left ventricular ejection fraction (LVEF) <35%, and left ventricular end-diastolic diameter (LVEDD) z-score >+4 with preserved right ventricular function. Primary endpoints were overall survival and transplant-free recovery, defined as sustained normalization of LV systolic function with reverse remodeling.

**Results:**

Fifty patients (27 female) met the inclusion criteria and underwent PAB at a median age of 188 days, 70% were in ROSS FC IV, and 42% were listed as urgent for HTx. DCM etiology was idiopathic in 42%, genetic in 22%, and myocarditis-associated in 36%. There was no procedural mortality. Overall survival was 90% and 72% achieved transplant-free functional recovery. Following PAB, magnetic resonance imaging–derived LVEF improved from 19% ± 7% to 56% ± 11%, and LVEDD z-scores decreased from +6.3 to +1 (*P* < .001). In 33 patients, recovery was sustained over a median follow-up of 7 years, including 15 patients with stable LV function for over 10 years.

**Conclusions:**

In infants and young children with severe LV-DCM, PAB is associated with high long-term survival, sustained transplant-free recovery, and durable reverse cardiac remodeling.

## Graphical Abstract

**Figure:**
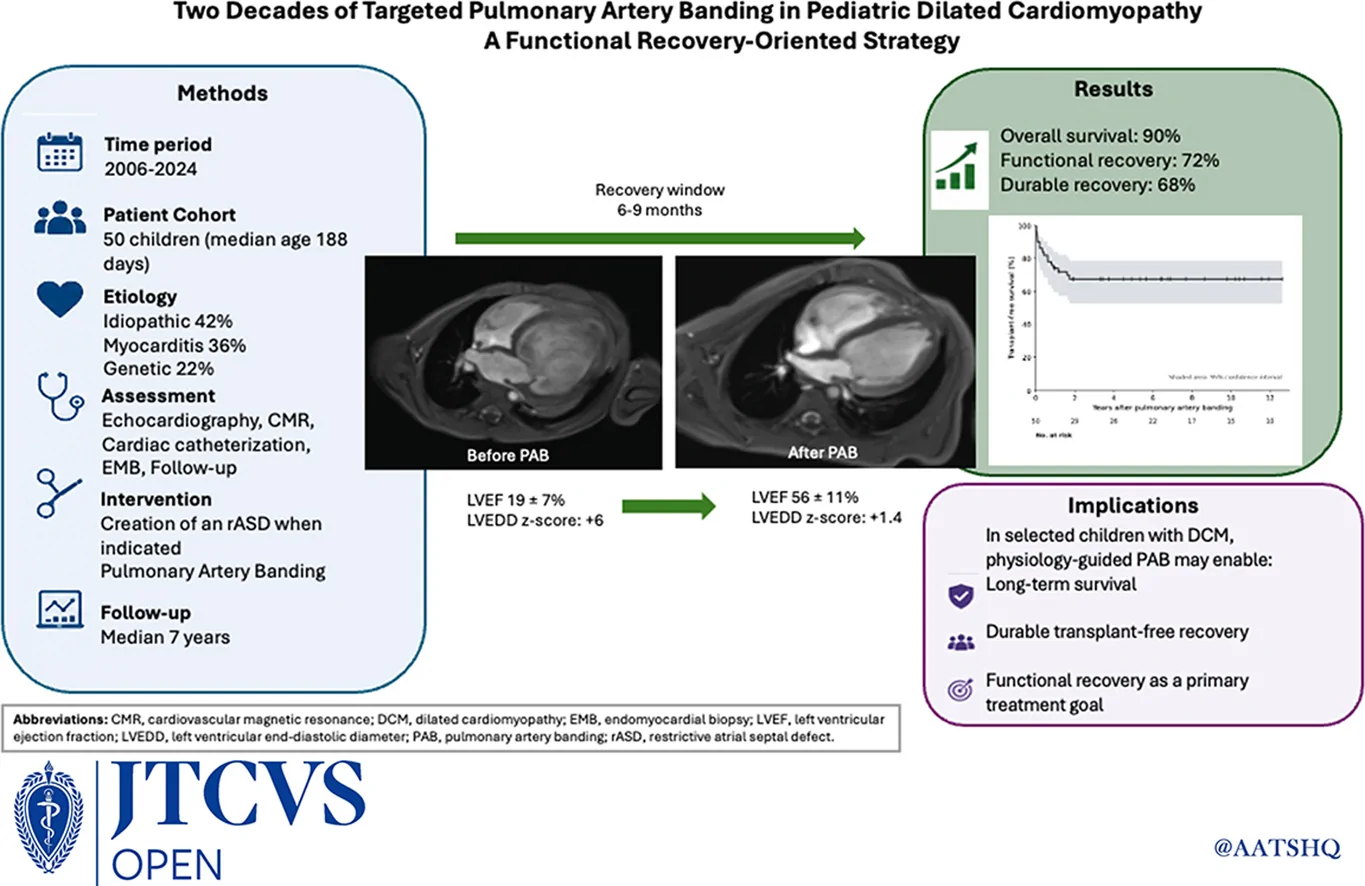

**Figure:**
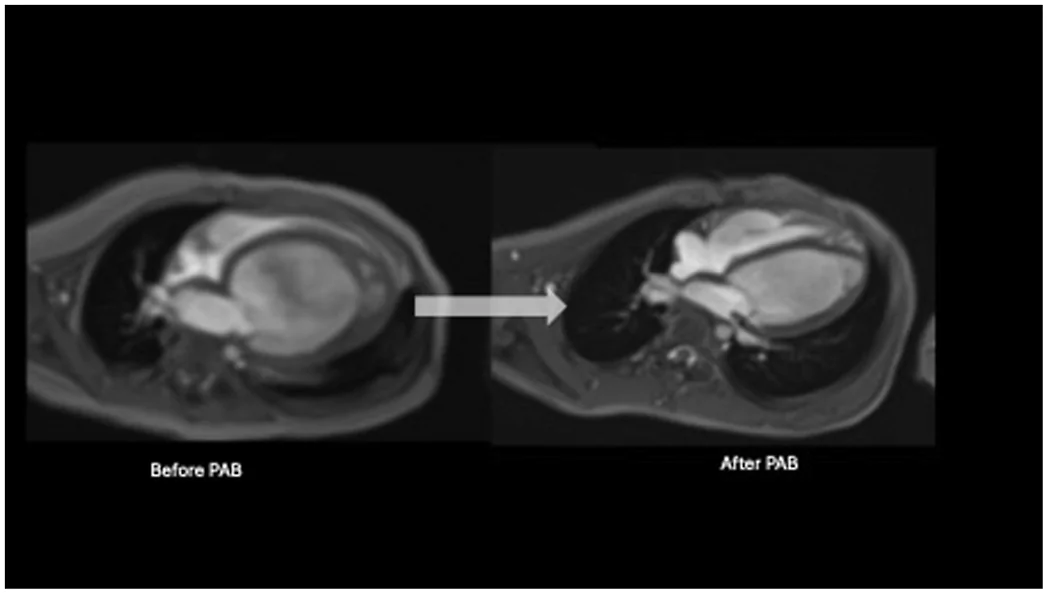
PAB supports durable reverse remodeling and transplant-free recovery in children with pediatric DCM.

**Central Message**

In selected infants and young children with severe dilated cardiomyopathy, pulmonary artery banding was associated with 90% survival and 72% transplant-free functional recovery, achieving durable reverse remodeling.

**Perspective**

A physiology-guided strategy combining rASD and PAB enabled durable functional recovery in the majority of infants with severe dilated cardiomyopathy, challenging the current paradigm of heart failure management and supporting the inclusion of this strategy in a treatment algorithm for selected patients.

Dilated cardiomyopathy (DCM) is defined by left ventricular (LV) or biventricular dilatation with systolic dysfunction in the absence of abnormal loading conditions or significant coronary artery disease.^1^ Pediatric DCM, although rare, represents a major cause of end-stage heart failure and cardiac-related mortality in infants and young children.^2^ At presentation, clinical severity ranges from asymptomatic disease to advanced heart failure and cardiogenic shock. Current heart failure therapies targeting sympathetic activation and the renin-angiotensin-aldosterone system can improve symptoms and outcomes,^3^ yet long-term prognosis remains poor in a substantial proportion of children with advanced DCM. Historical data indicate that nearly 40% of affected children die or undergo heart transplantation (HTx) within 2 years of diagnosis.^2^ Despite its low incidence, DCM remains one of the leading indications for HTx in this age group and frequently necessitates mechanical circulatory support (MCS).^4^

Importantly, complete recovery is considered as uncommon, particularly in patients with advanced disease and severe LV dilation.^5^ Data from the Pediatric Cardiomyopathy Registry indicate that only 22% of children with DCM achieve normal cardiac function within 2 years, with recovery more likely in younger patients and in those with less pronounced ventricular dilation. Even among patients achieving apparent normalization of cardiac function, relapse remains clinically relevant.^3^

Mechanical unloading with ventricular assist devices (VADs), combined with pharmacological therapy, may induce reverse remodeling, but sustained recovery seems unlikely, with only approximately 10% of pediatric patients successfully weaned from VAD support.^6^

These observations highlight the low likelihood of durable myocardial recovery in advanced pediatric DCM and the need to explore novel disease-modifying, recovery-oriented treatment strategies beyond conventional heart failure therapies.^7^

Nearly 2 decades ago, pulmonary artery banding (PAB) was introduced as a therapeutic strategy for infants and young children with advanced DCM listed for HTx.^8^^,^^9^ Mechanistically, PAB induces a leftward shift of the interventricular septum, reducing LV volume and reshaping LV geometry toward a more favorable Frank-Starling relationship. These changes are associated with increases in left ventricular ejection fraction (LVEF), reductions in left ventricular end-diastolic pressure (LVEDP), and improvements in electromechanical synchroneity. These clinical observations led to the hypothesis, later emphasized in an editorial by Leonard Bailey,^10^ that PAB may promote myocardial recovery by facilitating reverse remodeling in early childhood.

Given the intrinsic capacity of the immature myocardium for functional recovery,^11^^,^^12^ several centers have adopted PAB as a targeted intervention to modify ventricular mechanics and promote reverse remodeling.^13^, ^14^, ^15^ Despite these promising results, available data on PAB for treating DCM remain limited due to small sample sizes, heterogeneous inclusion criteria, variable diagnostic approaches, and limited follow-up duration, underscoring the need for refined phenotyping and long-term outcome data.^14^^,^^15^

Long-term durability of recovery, the role of staged transcatheter PAB dilatation, and outcomes after recovery remain insufficiently defined. We therefore analyzed 2 decades of institutional experience with PAB in infants and young children with severe DCM, focusing on survival, transplant-free recovery, and long-term stability after staged catheter-based PAB adjustment.

## Methods

This retrospective, single-center study included pediatric patients with DCM and severe heart failure who required inpatient treatment and underwent surgical PAB between 2006 and 2024 (Figure 1). All consecutive patients with the predefined inclusion criteria during the study period were screened. No strict upper age cutoff was predefined, but PAB was primarily intended for infants and young children with presumed myocardial plasticity. Over time, institutional practice favored patients younger than 3 years.

**Figure 1:**
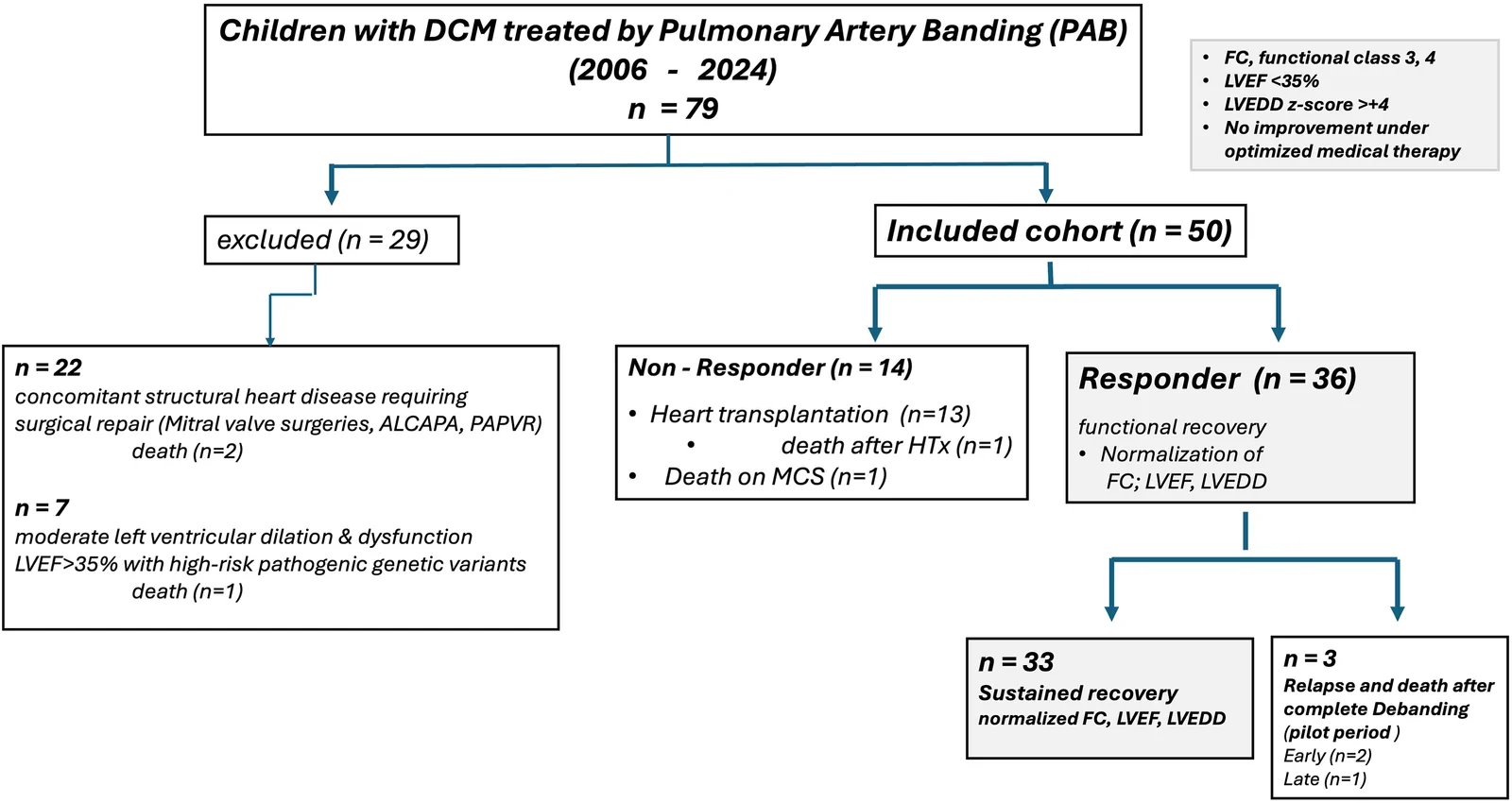
Study cohort selection and clinical outcomes after pulmonary artery banding. The flow chart depicts all patients undergoing pulmonary artery banding for left ventricular dysfunction and significant dilation between 2006 and 2024. Patients with concomitant structural heart disease or moderate left ventricular dysfunction were excluded. The included cohort comprised infants and young children with DCM and well-defined inclusion criteria and those who showed severe systolic dysfunction and marked left ventricular dilatation under optimized heart failure medication. *ALCAPA*, Anomalous left coronary artery from pulmonary artery; *DCM*, dilated cardiomyopathy; *FC*, functional class; *HTx*, heart transplantation; *LVEF*, left ventricular ejection fraction; *LVEDD*, left ventricular end-diastolic diameter; *MCS*, mechanical circulatory support; *PAPVR*, partial anomalous pulmonary venous return.

Of the 79 screened patients, 22 were excluded from a detailed outcome analysis due to relevant concomitant structural heart anomalies requiring additional surgical interventions (eg, mitral valve repair and partial anomalous pulmonary venous return repair). Seven patients with only moderately reduced LVEF (>35%) who underwent PAB because of a high-risk clinical profile (eg, proven pathogenic genetic variants with documented clinical disease progression) were also excluded. The final study cohort included 50 patients. Inclusion criteria were ROSS functional class (FC) III-IV, LV DCM phenotype with imaging-confirmed severely reduced systolic function (LVEF <35%), and marked LV dilatation (left ventricular end-diastolic diameter [LVEDD] z-score >+4) despite optimized medical therapy.

### Ethical Approval

The institutional PAB program was initiated in 2006 as a clinical treatment strategy. The retrospective analysis of clinical data was subsequently approved by the Ethics Committee of Justus-Liebig-University Giessen on November 20, 2013 (AZ 191/13), and the analysis of restrictive atrial septal defect (rASD)–related outcomes was approved on October 16, 2018 (AZ 198/18). The study was conducted in accordance with the principles of the Declaration of Helsinki, and written informed consent was obtained from the patients’ legal guardians, including consent for the use of pseudonymized data for scientific analysis and publication.

### Diagnostic Assessment and Treatment Strategy

Patient management followed a pathophysiology-guided, cardiac-recovery-oriented treatment algorithm aimed at promoting LV reverse remodeling and functional recovery (Figure E1).

Diagnostic assessment included baseline parameters, clinical severity, brain natriuretic peptide (BNP), echocardiography, cardiovascular magnetic resonance (CMR) imaging when feasible, invasive hemodynamics, endomyocardial biopsy (EMB) with viral analysis, and genetic testing when available. Because systematic PediMACS documentation was not available throughout the study period, an exact retrospective profile assessment was not performed.

Echocardiography was performed in all patients to assess LV systolic function, LV dimensions, mitral regurgitation, right ventricular (RV) function, and interatrial communication. CMR was used when clinically feasible for biventricular volumetry and functional assessment and tissue characterization. In patients without CMR, eligibility for PAB was determined by comprehensive echocardiographic assessment.

Cardiac catheterization was performed for invasive hemodynamic assessment and exclusion of coronary anomalies. In patients with elevated LV filling pressures and pulmonary venous congestion, transcatheter creation of a rASD or dilatation of a patent foramen ovale (PFO) was performed to provide left atrial decompression while maintaining restrictive communication. EMB samples were analyzed by histology, immunohistochemistry, and viral polymerase chain reaction. Genetic testing was performed when feasible, and variants were classified according to American College of Medical Genetics and Genomics criteria.

All patients received standardized heart failure therapy, including β1-blockade, an angiotensin-converting enzyme inhibitor or an angiotensin II type 1 receptor antagonist, and a mineralocorticoid receptor antagonist.

PAB was performed in patients with persistent LV dysfunction and dilatation despite hemodynamic stabilization and optimized medical therapy.

Detailed diagnostic, interventional, and surgical protocols are provided in the Appendix E1.

### Outcome Measures

Early improvement following PAB was defined as an increase in LVEF >40% alongside partial LV reverse remodeling (LVEDD z-score <+3). Full functional cardiac recovery was defined as normalization of LV systolic function and age-adjusted ventricular dimensions, defined as LVEF >55% and an age-adjusted LVEDD z-score <+2, confirmed by serial imaging during follow-up, and accompanied by clinical improvement to ROSS FC I. Transplant-free recovery was defined as functional recovery in the absence of death, HTx, or durable MCS.

### Statistical Analysis

Statistical analyses were performed using IBM SPSS Statistics (version 29.0, IBM Corp) and R (version 4.5.1, R Foundation for Statistical Computing). Categorical variables are presented as counts and percentages, and continuous variables are reported as mean ± standard deviation or median (interquartile range [IQR]), as appropriate, based on distribution.

Paired pre- and postintervention analyses were performed for patients with complete paired measurements for each endpoint using paired *t* tests or Wilcoxon signed-rank tests, as appropriate. Potential predictors of recovery were explored using univariable logistic regression. Overall and transplant-free survival were estimated using the Kaplan-Meier method. Overall survival was defined as the time from PAB to death, with patients censored at the last follow-up; transplant-free survival was defined as the time from PAB to death or HTx, whichever occurred first.

Analyses were performed using available data without imputation. All statistical tests were 2-sided, with a significance level set at *P* < .05.

## Results

### Patient Characteristics and Baseline Findings

Fifty infants and young children with severe DCM met the inclusion criteria and underwent surgical PAB. The cohort included 27 females (54%). Median age at PAB was 188 days (IQR, 107-440), with a median body weight of 6.3 kg (IQR, 5.3-8.7). No intraoperative mortality or procedure-related complications occurred.

All patients presented with advanced heart failure (ROSS FC III/IV), with 70% classified as FC-IV, were considered potential candidates for HTx. At admission, 35 patients (70%) required inotropic support; 15 patients (30%) were mechanically ventilated, and 12 patients (24%) had a history of cardiopulmonary resuscitation. Based on the available clinical data, most patients (70%) had advanced decompensated heart failure broadly corresponding to PediMACS 1-3. At presentation, 21 patients (42%) were listed for urgent HTx according to the local transplant allocation system. One patient required extracorporeal membrane oxygenation prior to PAB.

Baseline demographic and clinical characteristics are summarized in Table 1. Echocardiography was performed in all patients and demonstrated severely impaired LV function, with a mean LVEF of 21%±6.1% (n = 50) and marked LV dilatation (mean LVEDD, 45 ± 6.3 mm; mean z-score, 6.3 ± 1.4). Consistent with advanced heart failure, the median BNP concentration was markedly elevated at 2288 pg/mL (IQR, 665-4590).

**Table 1:** Clinical characteristics and baseline parameters of the study cohort prior to pulmonary artery banding

Baseline parameters	Number of patients N (%)	Median (IQR) Mean + SD
Study cohort	50	
Age at PAB, d		188 (107-440)
Weight at admission, kg		6.3 (5.3-8.7)
Female sex	27 (54)	
ROSS class IV	35 (70)	
Clinical severity before PAB		
Ventilation	15 (30)	
Inotropic support	35 (70)	
CPR	12 (24)	
High urgency listing	21 (42)	
ECMO	1	
Etiology∗		
Genetic DCM	11 (22)	
Idiopathic DCM	21 (42)	
Myocarditis	18 (36)	
LVNC	14 (28)	
Functional parameters		
LVEF, ECHO, %	50	21 ± 6.1
Mitral regurgitation > moderate	32 (64)	
LVEF, MRI, %	46 (92)	18 ± 6.7
RVEF, MRI, %	46 (92)	55 ± 12.7
LGE, positive	11 (22)	
Transmural LGE	2	
LVEDD, mm	50	45 ± 6.3
z-score LVEDD	50	
BNP, pg/mL	50	2288 pg/mL (665-4590)
Cath lab	46 (92)	
LVEDP, mm Hg		15 (12-20)
rASD creation or dilatation of PFO	21 (42)	
EMB		
Biopsy-proven myocarditis	18 (36)	
Fibrosis (focal/diffuse)	41 (82)	

*IQR*, Interquartile range; *SD*, standard deviation; *PAB*, pulmonary artery banding; *CPR*, cardiopulmonary resuscitation; *ECMO*, extracoporal membrane oxygenation; *DCM*, dilated cardiomyopathy; *LVNC*, left ventricular noncompaction; *LVEF*, left ventricular ejection fraction; *ECHO*, echocardiography; *MRI*, magnetic resonance imaging; *RVEF*, right ventricular ejection fraction; *LGE*, late gadolinium enhancement; *LVEDD*, left ventricular end-diastolic diameter; *BNP*, B-type natriuretic peptide; *LVEDP*, left ventricular end-diastolic pressure; *rASD*, restrictive atrial septal defect; *PFO*, patent foramen ovale; *EMB*, endomyocardial biopsy.

∗ Genetic testing was performed in 35 of 50 patients (70%).

### CMR and Invasive Assessment

CMR was performed whenever clinically feasible and was successfully obtained before intervention in 46 patients (92%), confirming the left ventricular-dilated cardiomyopathy (LV-DCM) phenotype. Mean CMR-derived LV-EF was 18% ± 6.7%. RV function was preserved in the majority of patients (mean RV-EF, 55% ± 12.7%), whereas 9 patients had reduced systolic function (mean RV-EF, 37.5% ± 5.55%), which improved to >45% under optimized therapy, including the creation of a restrictive atrial communication, making them eligible for PAB. Late gadolinium enhancement was detected in 11 patients (22%), including a transmural pattern in 2 patients. In patients without CMR prior to intervention (n = 4), eligibility for PAB was determined by a comprehensive echocardiographic assessment.

Invasive hemodynamic assessment was performed in 46 patients (92%) as part of the diagnostic workup, most commonly during preoperative hemodynamic evaluation before PAB (44/46, 96%). Median LVEDP was 15 mm Hg (IQR, 12-20). Creation of an rASD or dilation of a PFO was performed in 21 patients (42%), with elevated median LVEDP of 18 mm Hg (IQR, 13-20 mm Hg) and clinical or radiological signs of pulmonary venous congestion. Thirteen additional patients had a preexisting restrictive atrial communication and therefore did not require intervention.

Atrial decompression was achieved by stepwise balloon dilation under fluoroscopic and echocardiographic guidance, aiming to create a restrictive interatrial communication. After intervention, the median Doppler gradient across the interatrial communication was 5 mm Hg (IQR, 5-8 mm Hg). The median interval between catheter intervention (rASD creation/PFO dilatation) and PAB was 13 days (IQR, 6-37 days). During follow-up, repeat catheter intervention was required in 5 patients (24%) because of spontaneous closure or too excessive restriction of the rASD, resulting in recurrent pulmonary venous congestion. Procedural details, reinterventions, and complications are summarized in Table E1.

### Etiologic Classification and Histopathological Findings

A multimodal diagnostic approach integrating cardiac imaging, EMB, viral analysis, and genetic testing was used to identify the underlying etiology of DCM. Endomyocardial biopsies were obtained in 44 patients (88%). Interstitial myocardial fibrosis was present in 41 patients (82%). Biopsy-proven myocardial inflammation was identified in 18 patients (36%). Histopathological evaluation showed an acute/subacute pattern in only 4 patients (22%), whereas 14 patients had findings consistent with chronic inflammation. Among those with myocarditis-associated DCM, pattern of myocardial fibrosis was present in all cases, with a diffuse pattern in 13 patients. In addition, all patients had persistent severe LV dysfunction with marked LV dilatation and a clinical course >4 weeks before PAB, so they were classified as myocarditis with a DCM phenotype. Pathogenic or likely pathogenic genetic variants consistent with DCM were detected in 11 patients (22%). In 21 patients (42%), no definitive etiology could be identified, and these cases were classified as idiopathic. Irrespective of the underlying etiology, LV noncompaction morphology was observed in 14 patients (28%).

### Functional Recovery and Early Outcome

Following PAB, 36 patients (72%) achieved functional recovery, as documented by serial follow-up examinations. Under protocol-based, optimized heart failure therapy, the median heart rate decreased significantly from 140 bpm (IQR, 120-156) at baseline to 107 bpm (IQR, 99-114) at discharge (*P* < .001), with continuous improvement in ROSS FC (Figure E2). Among responders, early improvement—characterized by an increase in LVEF to >40% and partial reverse remodeling (LVEDD z-score, <+3)—was observed at a median of 9 months following PAB (IQR, 3-12). All responders were discharged home after a median hospital stay of 49 days (IQR, 34-90).

### Timing of Listing and Recovery

The swimmer plot (Figure 2) illustrates the temporal relationship between high urgency (HU) listing, early improvement, and delisting following PAB. HU listing frequently occurred before or shortly after PAB, reflecting the advanced heart failure severity at presentation. The swimmer plot further demonstrates the evolution of the listing strategy over time. During the early phase of the program, HU listing was initiated near PAB with short intervals to HTx. As institutional experience increased, listing was deferred to allow a time-dependent recovery window following PAB.

**Figure 2:**
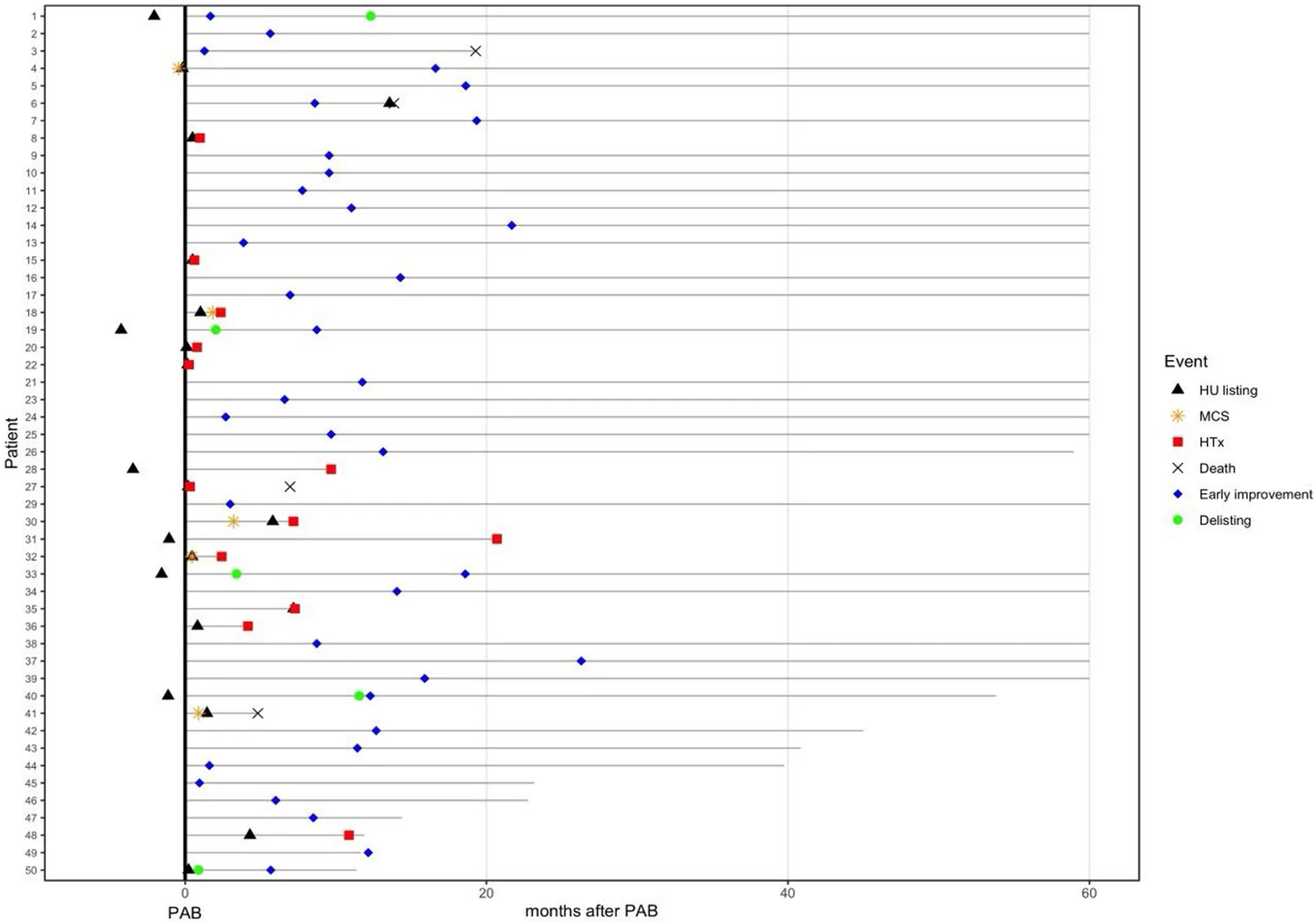
Swimmer plot illustrating the temporal relationship between HU listing, recovery, and adverse outcomes. Early improvement, defined as LVEF >40% and LVEDD z-score <3, was observed within 6 to 24 months after PAB. The graph also shows the evolution of the listing strategy: initially, HU listing occurred promptly before or immediately after PAB placement, with short waiting times for transplantation. *HTx*, Heart transplantation; *HU listing*, high urgency listing; *MCS*, mechanical circulatory support; *PAB*, pulmonary artery banding.

### Long-Term Outcome

During long-term follow-up (Table 2), patients who achieved functional recovery showed sustained ventricular reverse remodeling, with improvement in echocardiography-derived LV systolic function and normalization of the LVEDD (n = 36). Correspondingly, mean z-scores for LVEDD normalized from +6 to +1.4 (*P* < .001). In paired cardiac magnetic resonance imaging assessments (n = 33), mean LVEF increased significantly from 19% ± 7% to 56% ± 11% (*P* < .001), while CMR-derived right ventricular ejection fraction (RVEF) remained preserved (Figure 3, *A*-*D*). Consistent with structural and functional recovery, BNP levels declined from a median of 2679 pg/mL (IQR, 567-4799) to near-normal values of 36 pg/mL (IQR, 13-58) (*P* < .001) (Figure E2).

**Table 2:** Clinical outcomes and follow-up after pulmonary artery banding

Outcome	Number of patients (%)	Median (IQR) Mean + SD
Hospital stay, d	36	49 (34-90)
Recovery	36 (72)	
Maintained functional recovery	33 (66)	
Relapse	3	
2 early		
1 late		
Heart transplantation performed	13 (26)	
MCS after PAB	4 (8)	
3 bridging to HTx	3 (6)	
1 death on MCS		
Death	5 (10)	
3 with complete dilation		
1 on MCS		
1 after HTx due to graft failure		
Transcatheter PAB site dilatation strategy	36	
Complete dilation	3 (8)	
Partial dilation	30 (83)	
Time interval from PAB to partial dilation, mo		23 (9-39)
Overall survival	45 (90)	
Transplant-free survival	33 (66)	
Follow-up, y		7 (2-12)

*IQR*, Interquartile range; *SD*, standard deviation; *MCS*, mechanical circulatory support; *PAB*, pulmonary artery banding; *HTx*, heart transplantation.

**Figure 3:**
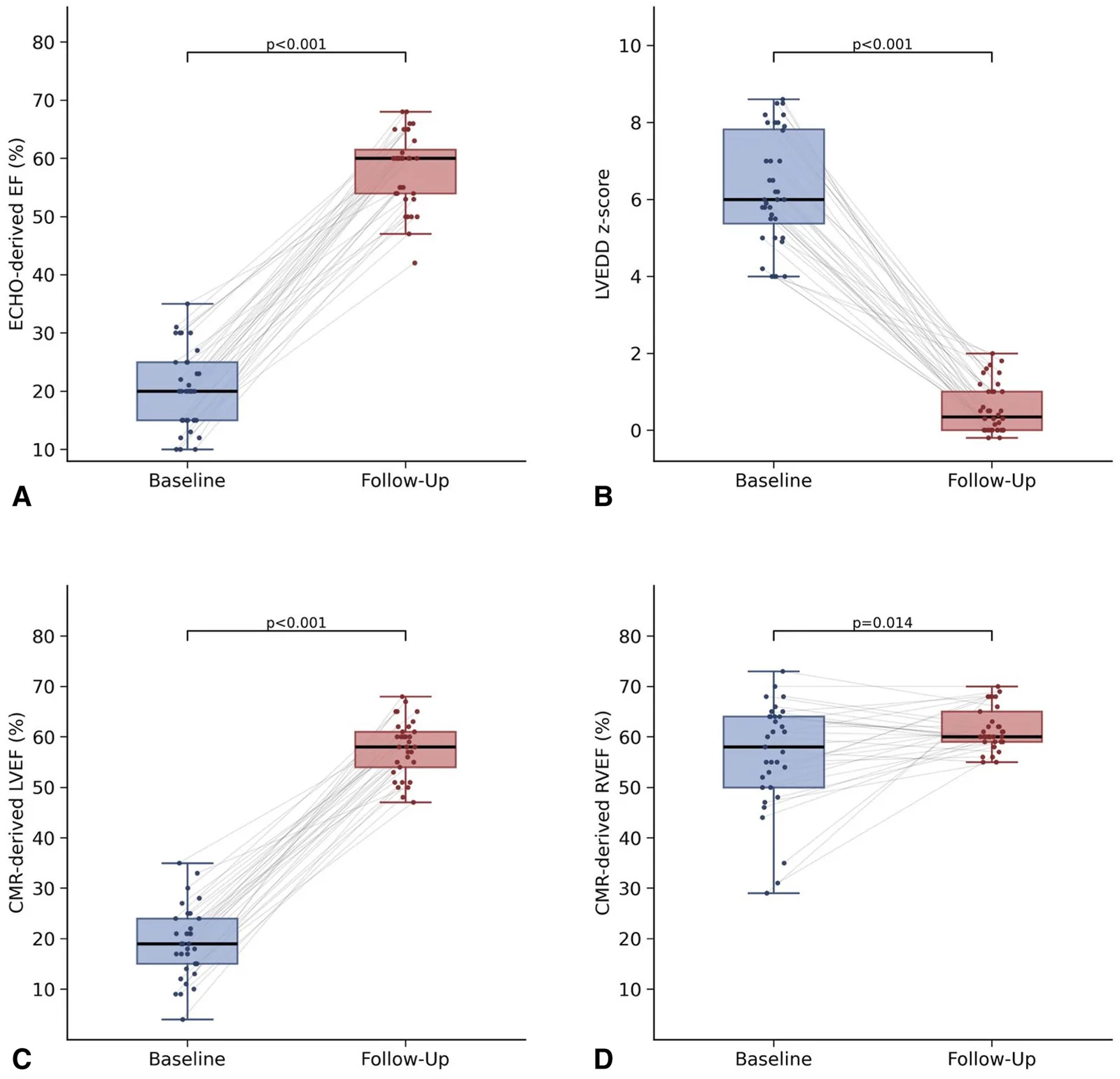
Ventricular reverse remodeling and functional recovery after pulmonary artery banding. Paired baseline and follow-up values are shown for echocardiography-derived LVEF (A), LVEDD z-score (B), CMR-derived LVEF (C), and CMR-derived RVEF (D). The *upper* and *lower borders* of the *box* indicate the upper and lower quartiles; the *middle horizontal line* represents the median; and the upper and lower whiskers show the maximum and minimum values in all box-and-whiskers graphs. *Dots* represent individual measurements; connecting lines indicate paired measurements. *P* values were calculated using paired pre-/postcomparisons. *CMR*, Cardiovascular magnetic resonance; *LVEDD*, left ventricular end-diastolic diameter; *LVEF*, left ventricular ejection fraction; *RVEF*, right ventricular ejection fraction.

### Durability and Failure of Recovery

Among the 36 patients who achieved functional recovery, 33 (92%) maintained preserved LV systolic function at the last follow-up (median, 7 years; IQR, 2-12). Three responders from the early pilot phase^9^ experienced relapse following complete transcatheter balloon dilatation and subsequently died after a median of 1.4 years following transcatheter balloon dilatation.

Based on this experience, the institutional strategy was modified to staged partial transcatheter balloon dilatation of the PAB site while maintaining a residual pulmonary artery (PA) gradient. Accordingly, 30 of the remaining 33 patients underwent partial balloon dilatation median of 24 months after PAB (IQR, 9-39), targeting a residual gradient of approximately 40 mm Hg (IQR, 35-40). RV function remained preserved during long-term follow-up after partial dilatation, with a mean RVEF of 61% ± 4.5%. Three patients remain under monitoring without further transcatheter intervention. Notably, 15 patients have maintained stable LV function for more than 10 years after partial balloon dilatation of the PAB.

MCS was required in 5 patients (10%) during the study period. Detailed patient-level information is provided in Table E2.

### Overall Survival and Transplant-Free Survival

Kaplan-Meier analysis showed an overall survival of approximately 90% at both 5 and 10 years after PAB. A single late death beyond 10 years in a patient from the early pilot phase after complete balloon dilatation led to a subsequent decline in the survival estimate. Transplant-free survival was 68% at 5 years and 65% at 10 years, with most events occurring early after PAB. (Figure 4, *A* and *B*).

**Figure 4:**
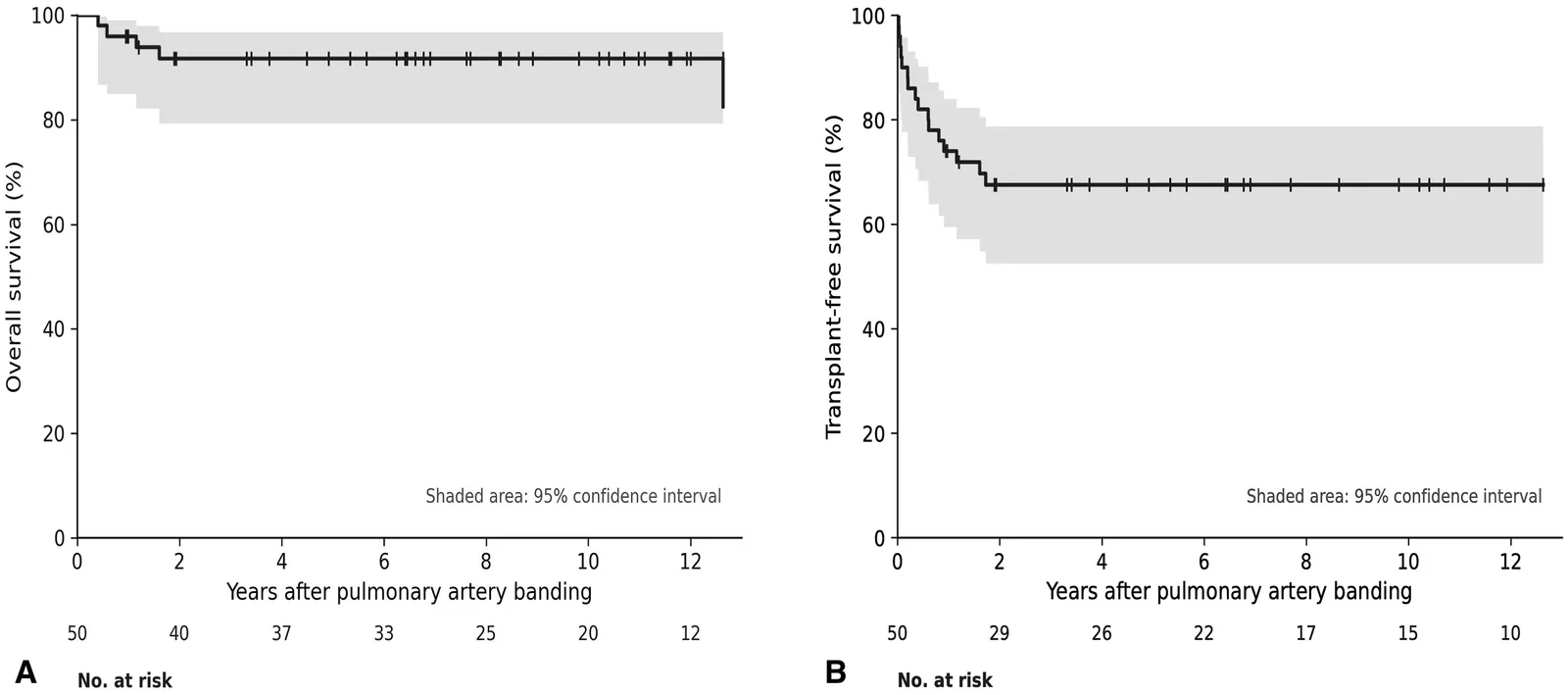
Overall survival and transplant-free survival after pulmonary artery banding. A, Kaplan-Meier estimate of overall survival of the study cohort (n = 50). Survival remained high throughout follow-up. B, Kaplan-Meier estimate of transplant-free survival, with events defined as death or heart transplantation. Most events occurred during early postintervention period. Event-free survival remained stable during long-term follow-up. Shaded areas represent 95% confidence intervals; numbers at risk are shown below each panel.

### Predictors of Recovery

Baseline clinical and imaging parameters did not differ significantly between responders and nonresponders with respect to age, sex, baseline ventricular function, LV dimensions, or fibrosis pattern. Genetic testing was available in 35 of 50 patients (70%). Genetic DCM was more frequent among nonresponders, whereas idiopathic DCM was more frequent among responders. However, transplant-free survival after PAB did not differ significantly across diagnostic categories, comprising myocarditis, idiopathic DCM, and genetic DCM (log-rank *P* = .525) (Table E3).

## Discussion

This study evaluates a physiology-guided PAB strategy in infants and young children (<5 years) with severe LV-DCM and advanced heart failure. Despite major advances in MCS, sustained transplant-free recovery in this age group remains uncommon.^4^ Moreover, in infants and young children, MCS is frequently associated with substantial morbidity and resource-intensive management, even when successful bridging can be achieved. Consequently, early bridging to HTx is often pursued, particularly when clinical, functional, morphological, histological, or genetic features suggest limited spontaneous recovery.^16^, ^17^, ^18^ However, young children with nonstructural cardiomyopathies retain a meaningful degree of age-related myocardial plasticity, providing a biological rationale for recovery-oriented strategies.^11^^,^^19^

Based on this concept, our approach integrates a multimodal diagnostic pathway with a stepwise, pathophysiology-driven hemodynamic strategy to create a time window for reverse remodeling and functional recovery.

### Multimodal Diagnostics and Patient Selection

A central determinant of success in this cohort was the use of a structured, multimodal diagnostic, and therapeutic approach. Comprehensive phenotyping enabled refined etiologic classification and risk stratification, distinguishing myocarditis, genetic cardiomyopathy, and idiopathic DCM.

Invasive hemodynamic assessment, particularly measurement of LVEDP, guided the selective creation of a restrictive atrial communication in patients with markedly elevated LV filling pressures. This intervention facilitated left atrial decompression, reduced pulmonary venous congestion, and likely preserved RV-PA coupling by improving RV filling.^20^

Transcatheter partial dilation of the PAB was performed once RV afterload exceeded the adaptive capacity while maintaining a residual pressure gradient. This staged approach appeared to support durable recovery and reduce late relapse, including in selected patients with genetic DCM.

These findings suggest that the efficacy of PAB is not attributable to a single factor but rather to a combination of diagnostic, hemodynamic, and therapeutic components.

### Mechanistic Interpretation of rASD and PAB

An important feature of the present strategy is the selective use of rASD for left atrial decompression in patients with elevated LV filling pressures. Beyond relieving pulmonary venous congestion, rASD creation may protect RV function by preventing secondary pulmonary venous hypertension, thus maintaining RV-PA coupling in a setting where PAB deliberately increases RV afterload.^20^ This step may expand eligibility for PAB by stabilizing the cardiopulmonary and ventricular-ventricular interaction prior to surgical afterload modulation. PAB reduces LV wall stress and induces a leftward septal shift, restoring pathological spherical LV geometry to a more physiological ellipsoidal configuration. These hemodynamic and mechanical changes may facilitate reverse remodeling by promoting ventricular-ventricular interaction during the period of increased myocardial plasticity.^19^^,^^21^

### Outcomes, Durability of Recovery, and Partial Balloon Dilatation Strategy

Among 79 children undergoing PAB without procedural mortality, detailed analysis of 50 infants meeting predefined functional criteria showed that a multimodal, recovery-oriented PAB strategy was associated with functional normalization despite advanced heart failure. Overall survival remained high at 90% at both 5 and 10 years, while transplant-free survival was 68% at 5 years and 65% at 10 years, with most events occurring early after PAB. Long-term stability was observed in 15 patients for more than 10 years after PAB. Escalation to MCS was required in only 10%.

Pathogenic or likely pathogenic variants were more common in nonresponders, indicating that genetics may affect recovery potential. However, some patients with confirmed genetic causes achieved durable stabilization.

### Transplantation Strategy and Recovery-Oriented Management

A further important observation was the evolution of the HTx listing strategy over time. Early in our experience, a substantial proportion of patients were listed for high-urgency transplantation before or shortly after PAB, whereas later practice allowed the monitored recovery window when clinically feasible. Notably, this shift toward deferred listing was not associated with an apparent increase in mortality, supporting the safety of a carefully monitored recovery-first strategy in selected patients. Similar concerns regarding premature transplantation have been raised in other pediatric heart failure cohorts.^22^ Contemporary registry data underscore the challenges of MCS in infants, which remains associated with substantial morbidity and low rates of myocardial recovery.^3^^,^^6^

### Study Limitations

This study has several limitations. Its retrospective single-center design and lack of randomization introduce potential selection and referral bias. Diagnostic approaches and management strategies evolved over the extended study period, which may have influenced patient selection, timing of intervention, and outcomes. An exact retrospective PediMACS assignment was not possible for all patients. Incomplete genetic testing means that an unidentified genetic substrate cannot be excluded in patients classified as idiopathic DCM. Repeat EMB was performed only selectively, mainly in myocarditis-associated cases, and was not available in sufficient numbers for paired histological assessment of reverse remodeling. Similarly, heart rate variability was not systematically analyzed across the full cohort; interpretation is further limited by concomitant ß1-blocker therapy and incomplete digital rhythm data in the earlier era. Furthermore, the generalizability of these findings beyond highly specialized centers with expertise in pediatric heart failure, invasive hemodynamics, and staged interventional strategies remains uncertain and requires external validation. Prospective multicenter studies applying standardized multimodal diagnostic and treatment protocols are needed to confirm the reproducibility of this strategy. Translational and molecular investigations are warranted to further elucidate these mechanisms.

## Conclusions

In infants and young children with severe DCM, a multimodal, pathophysiology-driven treatment strategy integrating transcatheter creation of a rASD and surgical PAB can enable sustained myocardial recovery, even in advanced disease stages of heart failure. When applied within a structured diagnostic and recovery-oriented framework, this approach may reduce the need for early transplantation and long-term mechanical support in carefully defined patients.

## Conflict of Interest Statement

The authors reported no conflicts of interest.

The *Journal* policy requires editors and reviewers to disclose conflicts of interest and to decline handling or reviewing manuscripts for which they may have a conflict of interest. The editors and reviewers of this article have no conflicts of interest.

## Figures and Tables

**Table E1:** Interatrial decompression procedures, reinterventions, and complications (n = 50)

Parameters	Number (%)	Median (IQR)	Details
Baseline parameters			
Number of total patient cohorts	50		
Invasive hemodynamic assessment performed	46 (92)		
Assessment performed before surgery	44 (96)		
Assessment performed externally before referral	7 (15)		
LVEDP	41 (89)	15 mm Hg (12-20 mm Hg)	
Preexisting PFO, no intervention	13 (26)		Considered sufficient for decompression
Interventions	21 (46)	18 mm Hg (13-20 mm Hg)	
Creation of rASD	15 (33)		
PFO dilatation	6 (13)		
Median time interval from intervention to PAB, d		13 (6-37)	
Median Doppler gradient after intervention, mm Hg		5 (5-8)	
Repeat catheter intervention during follow-up	5/21 (24)		
Repeat balloon dilatation	2 (10)		
Interatrial stent implantation	3 (14)		
Complications	5 (10)		
Procedure-related complications at external center	2		Resuscitation during catheterization
Procedure-related complications at the study center	1		Pericardial tamponade requiring treatment
Failed transseptal puncture	1		rASD creation performed during surgery (PAB)

*IQR*, Interquartile range; *LVEDP*, left ventricular end-diastolic pressure; *PFO*, patent foramen ovale; *rASD*, restrictive atrial septal defect; *PAB*, pulmonary artery banding.

**Table E2:** Mechanical circulatory support in the patient cohort (n = 50)

MCS characteristics	Number	Percentage	Details	Outcome
Patients requiring MCS	5	10		
Before PAB	1		ECMO before PAB, full functional recovery. Complete debanding with relapse 10 y later with MCS	Death on MCS while awaiting HTx
After PAB	4		Failure of recovery	3 underwent HTx, 1 died while on MCS
Additional details				
Genetically confirmed DCM	4/5			
HTx	3/5			
Death	2/5			

*MCS*, Mechanical circulatory support; *PAB*, pulmonary artery banding; *ECMO*, extracorporeal membrane oxygenation; *HTx*, heart transplantation; *DCM*, dilated cardiomyopathy.

**Table E3:** Baseline characteristics of responders and nonresponders to pulmonary artery banding in pediatric dilated cardiomyopathy

Baseline parameters	Responders	Nonresponders	*P* value
	n = 36	n = 14	
Age at admission, d	193 (IQR, 115-477)	177 (IQR, 103-321)	.689
Weight at admission, kg	6.3 (IQR, 5.6-8.7)	5.6 (IQR, 5-7)	.310
Gender (female)	19	8	1
ROSS class IV	22	10	.744
Inotropes	27	8	.304
Ventilation	11	3	
CPR	9	3	
Functional parameters			
LVEF, MRI, %	19 ± 7	17 ± 5	.411
RVEF, MRI, %	56 ± 11	50 ± 16	.277
Late gadolinium enhancement	9	2	
LVEDD z-score	6 ± 1.4	6 ± 1	.880
BNP, pg/mL	2679 (IQR, 567-4799)	2216 (IQR, 750-4500)	.803
Cath lab			
LVEDP, mm Hg	16 (IQR, 12-20)	13 (IQR, 12-21)	.504
EMB			
Biopsy-proven myocarditis	13	5	1
Diffuse myocardial fibrosis	30	12	1
Etiology/morphology			
Diagnosis			.270
Pathogenic/likely pathogenic mutation	7	7	.005
Noncompaction	11	3	.729

Continuous variables are presented as mean ± SD or median (IQR), as appropriate. Categorical variables are reported as n (%) values. *P* values are descriptive and reported for selected variables of clinical relevance. *IQR*, Interquartile range; *CPR*, cardiopulmonary resuscitation; *LVEF*, left ventricular ejection fraction; *MRI*, magnetic resonance imaging; *RVEF*, right ventricular ejection fraction; *LVEDD*, left ventricular end-diastolic diameter; *BNP*, B-type natriuretic peptide; *LVEDP*, left end-diastolic pressure; *EMB*, endomyocardial biopsy; *SD*, standard deviation.

**Figure E1:**
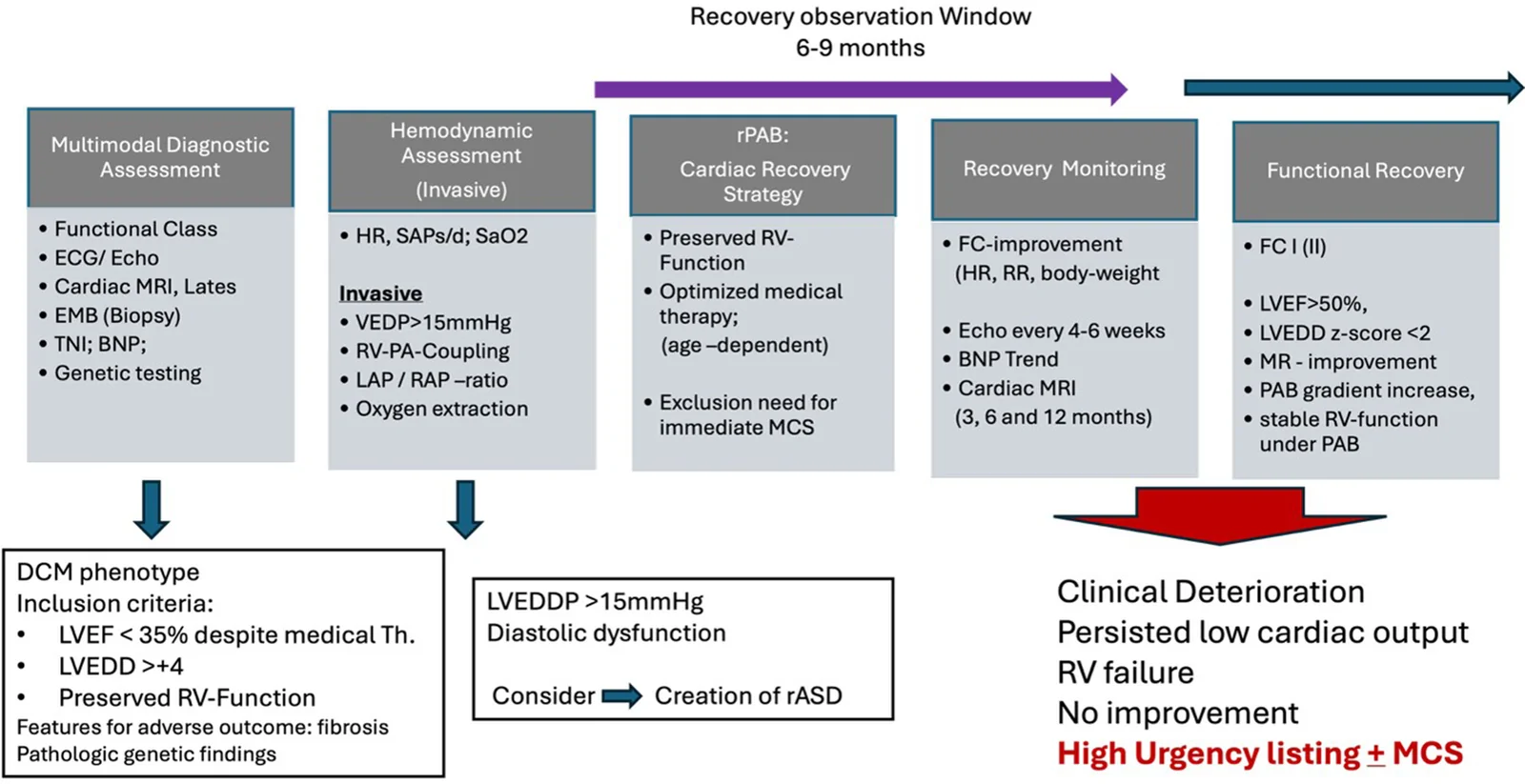
Pathophysiology-guided algorithm for recovery-oriented pulmonary artery banding in infants and young children. The algorithm integrates multimodal diagnostics and hemodynamic assessment and selectively creates a restrictive ASD in patients with elevated left ventricular filling pressures. PAB is implemented as a recovery-oriented intervention. There is a time-limited recovery observation after PAB, and recovery is assessed by clinical, biomarker, and imaging parameters. Clinical deterioration or lack of recovery prompts consideration of HU listing with or without MCS. *rASD*, Restrictive atrial septal defect; *BNP*, brain natriuretic peptide; *ECG*, electrocardiogram; *ECHO*, echocardiography; *EMB*, endomyocardial biopsy; *FC*, functional class; *HR*, heart rate; *LAP*, left atrial pressure; *LEVF*, left ventricular ejection fraction; *LVEDP*, left ventricular end-diastolic pressure; *MCS*, mechanical circulatory support; *MR*, mitral valve regurgitation; *MRI*, magnetic resonance imaging; *PAB*, pulmonary artery banding; *RAP*, right atrial pressure; *RV*, right ventricle; *SaO*_*2*_, systemic arterial oxygen saturation; *SAP s/d*, systolic arterial pressure systolic/diastolic; *TNI*, troponin I.

**Figure E2:**
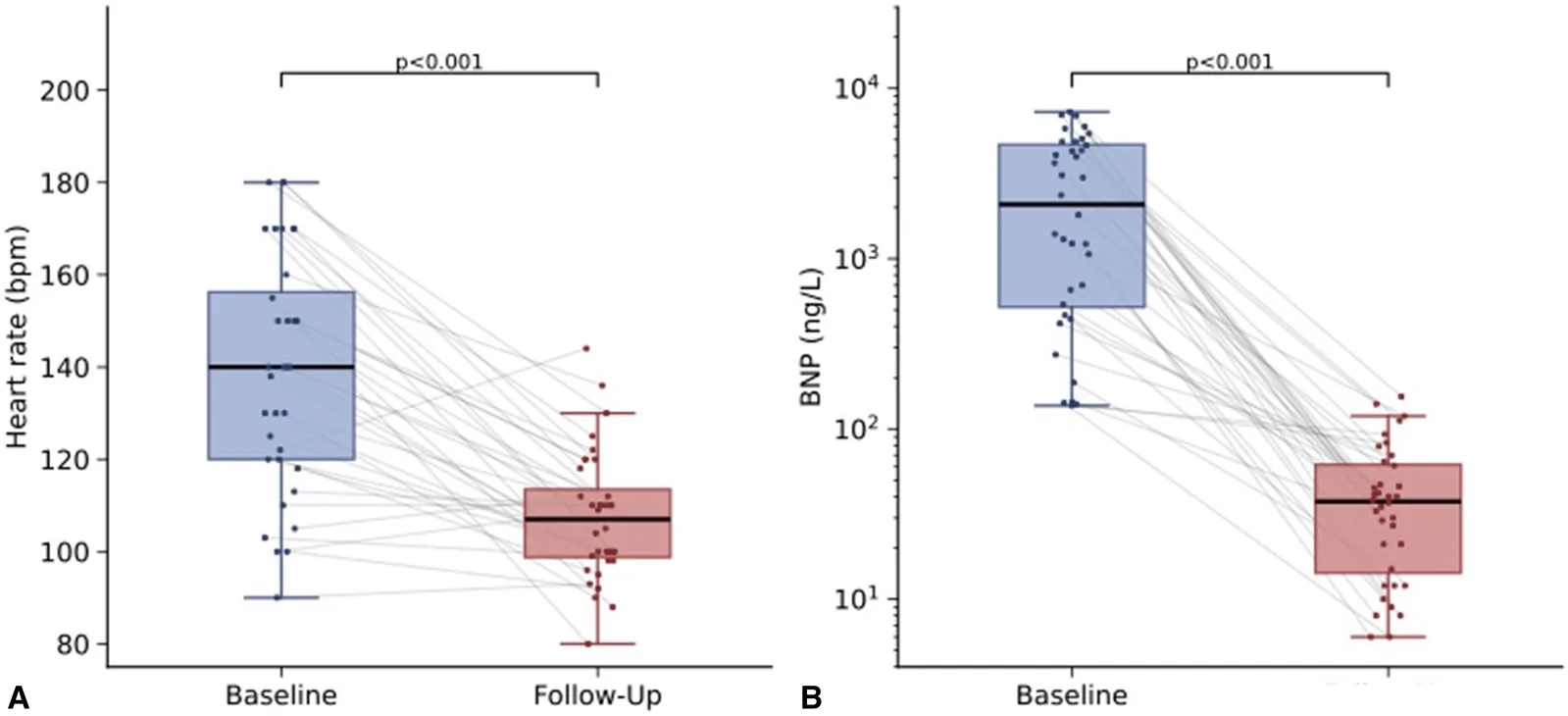
Clinical and biomarker response after reversible pulmonary artery banding. Paired baseline and postintervention values are shown for BNP (A) and heart rate (B). Heart rate was assessed at discharge; BNP was assessed at follow-up. Boxplots show median and interquartile range; whiskers indicate range. *Dots* represent individual patients, and connecting lines indicate paired measurements. BNP is displayed on a logarithmic scale. *P* values were calculated using paired pre/post comparisons. *BNP*, Brain natriuretic peptide.

## Appendix E1

### Clinical Assessment

Data were collected for the following baseline variables: age, sex, weight, height, and admission date. Heart failure severity was assessed using the modified ROSS classification, the pediatric analog of the NYHA functional classification, with classes I to IV representing increasing symptom severity and incorporating age-specific manifestations. Additional clinical parameters recorded included mechanical ventilation, inotropic support, and a history of resuscitation or mechanical circulatory support. Brain natriuretic peptide (BNP) levels were documented as a biomarker of heart failure severity.

### Echocardiography

Cardiac function was assessed using transthoracic 2-dimensional and color Doppler echocardiography. Left ventricular ejection fraction (LVEF) was determined using Simpson’s method. Left ventricular end-diastolic diameter (LVEDD) and corresponding z-scores were calculated using CardioZ (pediatric reference database, Evelina Children’s Hospital, London, United Kingdome). Right ventricular (RV) systolic function was evaluated by tricuspid annular plane systolic excursion. Due to severe left ventricular (LV) dilatation, asymmetric contraction, and altered ventricular geometry, fractional shortening was not considered a reliable measure of global systolic function.

The presence and hemodynamic characteristics of restrictive atrial communication or patent foramen ovale, including estimation of pressure gradients, were documented. Severe LV dysfunction was defined as an LV-EF of less than 35% despite medical therapy, as confirmed by echocardiography or cardiac magnetic resonance imaging (MRI). LV dilation, consistent with dilated cardiomyopathy (DCM), was defined as an LVEDD z-score >+2, with severe dilatation characterized by an LVEDD z-score >+4. Mitral valve regurgitation was categorized in 3 grades; structural mitral valve disease was excluded.

### Cardiac MRI

The MRI was performed using a 3-Tesla MRI system (MAGNETOM Verio, Siemens Medical Systems). The protocol included LV and RV volumetry, functional assessment, and tissue characterization using T1- and T2-weighted imaging. Furthermore, late gadolinium enhancement (LGE) was used: LGE results from regional differences in myocardial extracellular volume and from varying uptake and washout patterns within the extracellular space and is seen in myocardial injury (eg, myocyte necrosis, edema, myocardial scar tissue, and focal areas of fibrosis). MRI-driven parameters were used for the analysis; echocardiographic LVEF was used when MRI was not feasible, particularly during the early study period. Right ventricular function was primarily assessed by MRI, and RV function was classified as normal (right ventricular ejection fraction [RVEF] >55%) and mildly reduced (45%-54%). Preserved or improved RV function for PAB was defined as RVEF ≥50%; in selected cases, 45% to 50% was accepted.

### Cardiac Catheterization and Endomyocardial Biopsy

Cardiac catheterization was performed for invasive hemodynamic assessment and to exclude coronary anomalies. In elective patients, the procedure was performed under sedation with spontaneous breathing maintained. Transcatheter creation of a restrictive ASD (rASD) was usually performed using the Brockenbrough technique, as previously described.^E1^ A high-pressure balloon was advanced through the initially placed 6- or 8-Fr transseptal sheath whenever technically feasible. The interatrial septum was dilated stepwise with increasing balloon diameters to create an interatrial communication, as desired. The procedure was guided by assessment of defect size, Doppler flow pattern, and the interatrial pressure gradient. In general, interatrial septal dilation was considered complete when the balloon-to-target atrial septal defect diameter ratio was approximately 2:1. Echocardiography confirmed a stable restrictive communication. In cases of recurrent restriction or closure during follow-up, repeat balloon dilatation or implantation of an interatrial stent was considered to maintain this communication.

Endomyocardial biopsy samples were collected from the right ventricular septum and analyzed histologically and immunohistochemically. Polymerase chain reaction testing was conducted to detect viral genomes.

### Histopathology and Immunohistochemistry

Tissue samples were analyzed using conventional histology, immunohistology, and molecular diagnostics. Acute lymphocytic myocarditis was defined as the presence of more than 25 lymphocytes per mm^2^, along with an increased number of CD68+ macrophages. Myocarditis subtype was classified according to the modified Dallas criteria.^E2^ Fibrosis patterns consistent with advanced remodeling, including interstitial and replacement fibrosis, were assessed and categorized as focal or diffuse.^E3^

### Genetic Testing

Next-generation sequencing was performed when feasible to identify pathogenic variants associated with cardiomyopathy. A custom gene panel (Twist Bioscience) was used to cover all DCM-associated genes with definitive, strong, moderate, and limited evidence.^E4^ Variants were classified according to the most recent American College of Medical Genetics and Genomics guidelines.

### Medical Treatment Regimen

All patients received standardized heart failure therapy based on a triple neurohumoral blockade strategy, including selective β1-adrenergic blockade, long-acting angiotensin-converting enzyme inhibitors or angiotensin II type 1 receptor antagonists, and mineralocorticoid receptor antagonists. Continuous inotropic treatment was used as needed; milrinone and levosimendan were preferred. If catecholamine treatment with epinephrine was initiated, it was usually protected by concomitant β1-selective adrenergic blockade, typically via continuous infusion of esmolol or landiolol during the intensive care course. Detailed perioperative and medical management strategies have been previously reported.^E5^

### Surgical Pulmonary Artery Banding

Surgical pulmonary artery banding (PAB) was performed in patients with persistent, severe LV dysfunction and dilatation, despite hemodynamic stabilization and optimized medical therapy, in accordance with our institutional protocol. Open-chest surgery was performed via median sternotomy and limited pericardiotomy. A polytetrafluorethylene band was placed around the main pulmonary artery (PA) and adjusted under stable hemodynamic conditions using direct invasive RV pressure measurement obtained via an arterial line placed in the RV, with continuous invasive systemic arterial pressure and central venous pressure monitoring, RV function assessment, and ventricular septal leftward shifting monitored through transesophageal echocardiography guidance. The aim was to achieve a right ventricular systolic pressure of approximately 40% to 60% of the systolic systemic arterial pressure, while avoiding a significant rise in central venous pressure and systemic hypotension. The band adjustment was performed using 2 double sutures, allowing subsequent transcatheter partial dilatation. Perioperative and postoperative management followed standardized institutional protocol as previously described.^E6^ Although minor experience-based refinements occurred over the study period, the underlying concept, the strategy, and the PAB target settings remained largely consistent throughout the study period.

### Follow-up

Follow-up examinations were performed according to our institutional protocol and included regular clinical assessment, ECG, echocardiography, and serial BNP measurements. Follow-up visits were scheduled at close intervals during the first 12 months after PAB and were subsequently adapted according to clinical status, ventricular function, and the degree of reverse remodeling.

Cardiac magnetic resonance (CMR) imaging was planned within the first 6 months after PAB, usually at 3 to 6 months, followed by repeat CMR at approximately 12 and 24 months. Thereafter, it was repeated at 2 to 3 years whenever feasible. For the present analysis, CMR data were evaluated at predefined timepoints, including baseline (pre-PAB), the time point of relevant improvement, and the last available CMR during follow-up. The interval between PAB and the last available CMR during follow-up is reported to describe the duration.

BNP/NT-pro-BNP levels were closely monitored during the first year after PAB and were subsequently assessed during follow-up, mainly in conjunction with CMR examinations. Repeat endomyocardial biopsies were not performed routinely but were reserved for selected cases, especially those with myocarditis (to assess the grade of inflammatory activity).

### Transcatheter PA Balloon Dilatation

Percutaneous transcatheter partial balloon dilatation with gradual dilation of the PAB was considered when relevant pressure overload of the right ventricle occurred. Typical criteria included a Doppler-derived pressure gradient across the PAV of 60 to 70 mm Hg, progressive RV hypertrophy, and dilatation of the RV with increasing tricuspid regurgitation. PAB dilatation was performed using a high-pressure balloon. Balloon size was selected based on the narrowest PAB diameter measured during the angiographic assessment. Additionally, the pulmonary valve anulus and the main proximal PA diameter proximal and distal to the band were measured, preferably in a 90° lateral projection. Ballon diameters were increased stepwise. Based on our initial experiences, the primary goal was usually to achieve a moderate pressure gradient across the residual PAB of 35 to 45 mm Hg, while complete balloon dilatation was not performed, especially in genetically confirmed cardiomyopathy and in morphologic NC cardiomyopathy.
